# Supplementary material for: Exploiting horizontal pleiotropy to search for causal pathways within a Mendelian randomization framework
Source: Nat Commun. 2020 Feb 21;11:1010. doi: 10.1038/s41467-020-14452-4 (PMC7035387; doi:10.1038/s41467-020-14452-4)
Supplement: Supplementary file 6 — Description of Additional Supplementary Files [file 41467_2020_14452_MOESM6_ESM.pdf]

Title: Supplementary Data 1. MR of candidate traits on each outcome.

Description: SNP, single nucleotide polymorphism; N SNPs, number of SNPs; SE, Standard error; VLDL, very low-density lipoprotein; HDLC, high density lipoprotein cholesterol; LDLC, low density lipoprotein cholesterol; CI, confidence interval. The number of SNPs used for two sample MR analysis of candidate traits on the outcome. The results were presented as IVW beta coefficient (95% CI), derived from two sample MR analyses.

Title: Supplementary Data 2. Proportion of unbiased estimates and AUROC value across different scenarios and methods.

Description: The top panel of the table presents the proportion of simulations that lead to unbiased effect estimates of X on Y. The bottom panel of the table presents the ability (proportion) of the method to distinguish between simulations in which the causal effect of x on y is either null or not null. 'Raw’ = IVW random effects estimates applied to all detected instruments; ‘Removed’ = either all outliers are removed, or only outliers detected to associate with a candidate trait; ‘MVMR’ = multivariable MR using either candidate traits detected to associate with any instrument or using only candidate traits associated with outlier instruments; ‘Adjusted’ = Adjusting SNP-outcome associations for candidate traits applied either only to variants detected to be outliers, or all variants regardless of outlier status.
